# Evaluation of *In Vivo* Acaricidal Effect of Soap Containing Essential Oil of *Chenopodium ambrosioides* Leaves on *Rhipicephalus lunulatus* in the Western Highland of Cameroon

**DOI:** 10.1155/2015/516869

**Published:** 2015-12-06

**Authors:** Marc K. Kouam, Vincent K. Payne, Emile Miégoué, Fernand Tendonkeng, Jules Lemoufouet, Jean R. Kana, Benoit Boukila, E. Tedonkeng Pamo, Bertine MNM

**Affiliations:** ^1^Department of Animal Production, Faculty of Agronomy and Agricultural Sciences, University of Dschang, P.O. Box 222, Dschang, Cameroon; ^2^Center for Research on Filariasis and Other Tropical Diseases (CRFilMT), P.O. Box 5797, Yaoundé, Cameroon; ^3^Department of Animal Sciences, Faculty of Sciences, University of Dschang, P.O. Box 67, Dschang, Cameroon; ^4^Higher National Institute of Agronomy and Biotechnology (INSAB), University of Sciences and Techniques of Masuku, P.O. Box 941, Franceville, Gabon

## Abstract

A study on the acaricidal properties of foam soap containing the essential oil of *Chenopodium ambrosioides* leaves was carried out on *Rhipicephalus lunulatus*. Four doses (0.03, 0.06, 0.09, and 0.12 *µ*L of essential oil per gram of soap) and a control (soap without essential oil) with four replications for each treatment were used for *in vitro* trial. Each replication consisted of 10 ticks in a Petri dish with filter paper impregnated with the foam soap on the bottom. Following *in vitro* trials, three doses (0.06, 0.09, and 0.12 *µ*L/g) and the control in two replications were selected for *in vivo* test based on mortality rate recorded from the *in vitro* trial. Each replication was made up of 10 goats naturally infested with ticks. Results show that soap containing essential oil is toxic to *R. lunulatus*. The *in vivo* mortality rate in the control on day 8 was 22.69% whereas the highest dose (0.12 *µ*L/g) killed 96.29% of the ticks on day 8. The LD_50_ of the foam soap containing essential oil was 0.037 and 0.059 *µ*L/g on day 2 in the laboratory and on the farm, respectively. This indicates the potentially high efficiency of this medicated soap on this parasite.

## 1. Introduction

Ruminant breeding constitutes one of the main production activities in many sub-Saharan African countries in general and Cameroon in particular [1]. Although small ruminants in Cameroon have attracted interest as an alternative and cheap source of income and protein, they are also exaggeratedly used during festivities and traditional rites. Based on these considerations, goats in particular represent for breeders an easily mobilizable investment due to their short developmental cycle [2]. Data on the importance of goat industry in the country is very scarce, but globally goats represent 20% of ruminant production in developing countries [3]. Because goats' breeding especially depends on natural pasture for their diet, normally they are exposed to a wide variety of constraints, some of which affect growth and quality of mutton. Their constant exposure to natural pastures persistently exposes them to arthropods most especially ticks which in the course of feeding on blood could also transmit disease. Indeed ticks do not only transmit diseases such as piroplasmosis, cowdriosis, or rickettsiosis [4] but also cause skin lesions, asthenia, and anorexia leading to anemia [5]. Ticks in general and* Rhipicephalus lunulatus* in particular constitute one of the main causes of morbidity and mortality in animals [6] and are also responsible for secondary pathogen infections [7].* R. lunulatus *is a two-host tick; larva and nymph feed on the same host, and adult feeds on a second host on which it remains for five to nine days.

Losses incurred worldwide due to ticks' infestation are enormous and even incalculable [8]. According to some authors [9], tick control under large-scale production system is through the use of acaricides. On the other hand, while poor local breeders control these ticks by manual removal, others still apply medicinal plants or use both methods. The large-scale application of organophosphates, carbamates, and organochlorines is a profitable venture which has yielded positive results [9]. Despite their high cost and environmental unfriendliness to man, animals and other nontarget organisms make the increasing need for alternative measures of tick control imperative.

Thus it seems advantageous to use natural substances like essential oil having therapeutic properties, extracted from some plants previously studied. Besides, there are reports that some of these essential oils have biological properties, some of which are acaricidal, antipyretic, bactericidal, insecticidal, antiseptic, and anti-inflammatory [10, 11].

The leaves of* Chenopodium ambrosioides*, widely found in tropical and subtropical regions of America and Africa [12], have been documented to contain essential oils that are both irritating and toxic [12, 13]. This study was therefore aimed at finding an efficient, cheap, sustainable, and easily applicable method of using essential oils to fight against ectoparasites in general and ticks in particular.

## 2. Material and Methods

### 2.1. Study Area

The study was carried out in the Western High Lands of Cameroon, between 25° and 6° north latitude and between 10° and 11° east longitude. The mean altitude of the region is 1420 m. The climate is equatorial. In this zone, rainfall varies between 1500 and 2000 mm/year. Annual temperatures vary between 10°C in July and 25°C in February. There are two main seasons in the region: a short dry season running from mid-November to mid-March and a long rainy season (corresponding to cultural season) from mid-March to mid-November [14]. Subsistence agriculture, together with breeding and trade, is the main economic activity of the region. The vegetation is the savannah with shrub and sparse forests in some areas [15].

### 2.2. Extraction of Essential Oils of* C. ambrosoides*

Leaves of the plant were harvested, taken to the laboratory, and dehydrated for three days at room temperature before extraction. Leaves of the plant were harvested before flowering between April and May in the rainy season. Oil was extracted by the hydrodistillation technique [10] using modified Clevenger type apparatus. The technique consisted in placing the mixture of plant and water on a hot plate for eight hours. The essential oil was obtained through evaporation. The mixture of oil and water vapor condensed in the distillation apparatus, where cool water circulates permanently. A two-layered distillate with the upper part being the essential oil was collected into a graduated cylinder tube associated with the distillation apparatus. After allowing water to flow out through a tap, the oil was then collected in a container and dehydrated using anhydrous sodium sulfate. The calculation of the yield was done using the following formula:

(1) $$\begin{array}{c} \text{Yield}\left(\;\%\;\right)=\frac{\text{weight of essential oil}}{\text{weight of the plant material}}\times 100. \end{array}$$

The chemical composition of the oil, as previously described by Tapondjou et al. [12], is as follows: *α*-terpinene (37.6%), Cymol (P-cymene) (50, 0%), cis-*β* farnesene (1.4%), ascaridole (3.5%), and carvacrol (3.3%). The plant used in this study and the one used by Tapondjou et al. [12] were both harvested in the same site and season.

### 2.3. The Production of Soap

Solutions of soda and Sikalite were mixed in a container and allowed to stand for 15 minutes. After this, palm oil was added and mixed by turning the preparation in the same direction for 15 minutes without resting during which essential oil was added. The final mixture was then put into appropriate moulds and left on the ground in darkness for seven days for solidification.

### 2.4. *In Vitro* Tests

Ticks used for* in vitro* tests were collected from African dwarf goats in villages of the Western High Lands of Cameroon. The harvesting was done manually without any distinction of sexes and with great care, avoiding the destruction of their rostrum. Ticks were weighed on a scale of the type “Denver Instrument” with a capacity of 210 g and a sensitivity of 0.001 g. Their length was also measured using a millimeter paper. The average weight and length were 0.05 ± 0.01 g and 6.5 ± 0.04 mm, respectively. Selected adult ticks were now fixed with ethyl acetate and identified as* R. lunulatus* as previously described [16]. Once identified, ticks (adults) were ready for the tests. Ticks were fixed just to ease the identification process. Tick specimens used for* in vitro* and* in vivo* tests were not fixed.

The soap produced for bioassays had a weight of 450 g and contained 900 *μ*L of essential oils, that is, a dose of 2 *μ*L/g. To obtain applicable concentrations for* in vitro* tests, several dilutions of soap were made to find the most efficient concentration. The following doses were selected: 0.03; 0.06; 0.09; and 0.12 *μ*L/g of soap. Final doses to use on the farm for* in vivo* tests were obtained after a series of tests* in vitro.* Three of the four doses, the highest ones (0.06, 0.09, and 0.12 *μ*L/g), were selected. For both* in vivo* and* in vitro* tests, soap without essential oil was used as control.

Tests consisted of the evaluation of* in vitro* toxicity by contact of soap foam on ticks. The doses obtained above were applied to ticks. With a 10 mL pipette, the solution was uniformly distributed in Petri dishes with an area of 63.61 cm^2^, in which a round filter paper (Type Whatman number 1 with a diameter of 9 cm) had already been placed. Each treatment had four replicates made of 10 ticks introduced in one of the above Petri dishes. Counting of dead ticks was done every 24 hours for eight days. The mortality rate in each dish was calculated following the Abott method [17] according to the following formula:

(2) $$\begin{array}{c} M_{c}=\frac{\left(M_{0}-M_{e}\right)}{\left(100-M_{e}\right)}, \end{array}$$

where  *M*_0_ is mortality of ticks registered in treated replicate, *M*_*e*_ is mortality of ticks in the control, and *M*_*c*_ is corrected mortality of ticks (%).

The LD_50_ was determined by the Bliss method (1938) as redescribed by Valette [18] based on the regression of mortality depending on the logarithm of essential oil doses. This method was used for both* in vitro* and* in vivo* tests.

### 2.5. *In Vivo* Tests

Host animals chosen based on the infestation level with ticks were grouped into four sets of 10 goats each (i.e., 40 goats) in which three were treated with soap containing essential oil, and the other one, the control, was treated with soap without essential oil. Each set (four sets in total) was made up of 10 goats randomly selected, irrespective of the number of ticks each goat carried, which varied from 10 to 20. Each of the four sets was submitted to one dose of essential oil treatment mentioned above (i.e., soap without essential oil (control), soap with essential oil at doses of 0.06, 0.09 and 0.12 *μ*L/g of soap). Each replicate representing a dose of oil was done in two replicates (the treatment was applied on each animal two times a day: early in the morning and in the evening to improve the efficiency of the treatment). Ticks on the animals were counted before the onset of the tests. The test consisted in applying lather on the animal, focusing on points where ticks were susceptible to group. After the treatments, mortalities were evaluated every 24 hours for eight days. Host animals used were those that, during the past two months before the test, had not been submitted to any drug or specific chemical compound that could interfere with the results.

### 2.6. Statistical Analysis

Data obtained were analyzed using ANOVA [19] after correction of observed mortalities in relation to those of the control, and the differences between treatments when they existed were separated at 5% significance level by Student's *t*-test.

## 3. Results and Discussion

### 3.1. *In Vitro* Effects of the Essential Oil-Based Soap from* C. ambrosoides* Leaves on* R. lunulatus*

The yield of the oil extraction was 0.024%. This was lower than the results reported by Quarles [20] and Tapondjou et al. [12], which were 0.4% and 0.8%, respectively. This difference can be explained by many factors including method of distillation and the period during which the plant was harvested. Indeed, hydrodistillation can sometimes lead to loss of some quantity of essential oil during purification, while plant harvested in dry season can produce more oil than in rainy season. Besides yield the other antitick factors normally vary with season of collection and method of extraction and hence efficacy of two different extracts collected during different seasons cannot be compared.

**Figure 1:**
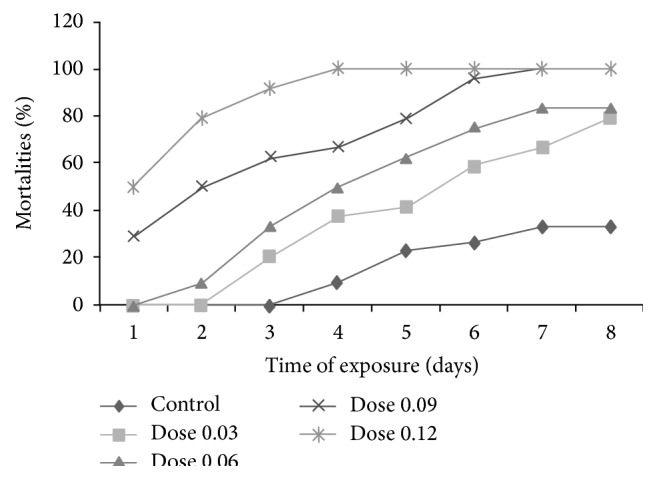
Evolution of* in vitro* mortalities according to time and doses of essential oils of* C. ambrosoides* leaves contained in the soap.

The evolution of mortalities in time depending on the doses of the essential oil incorporated in the soap is illustrated in Figure 1.

From this figure, the mortality rate of* R. lunulatus* increased both with the doses of essential oil in the soap (*μ*L/g) and with time. This mortality was maximal (100%) on the fourth and the seventh days, respectively, for the doses 0.12 and 0.09 *μ*L/g. For the doses 0.03 and 0.06 *μ*L/g, the highest mortality on the 8th day was 79.16% and 83.33%, respectively. The mortality remained relatively weak in the controls (33.33% on the 8th day). The mortalities observed in controls may be attributed to soap ingredients and particularly soda, though there is also a possibility of the occurrence in soap of other chemicals not yet known and reported. The difference in mortality between treated replicates and the control showed the toxicity of essential oil of* C. ambrosoides* leaves contained in soap on* R. lunulatus*.

The toxicity of essential oil may have been partly due to a local irritation of the digestive tube of the parasite and even more by the direct repressive action on its cardiovascular and respiratory systems [20]. This toxicity is generally attributed to ascaridole [12]. Symptoms of ascaridole poisoning include nausea, depression, and extreme fatigue in human [20] which could be due to a possible nervous system attack. Thus, this substance may have had some effects on the nervous system of* R. lunulatus*. According to Tapondjou et al. [12], toxicity of these oils can also be attributed to other major compounds such as thymol and *α*-terpinene. These compounds are known for their insecticide and acaricide effect [12, 21–23].

**Figure 2:**
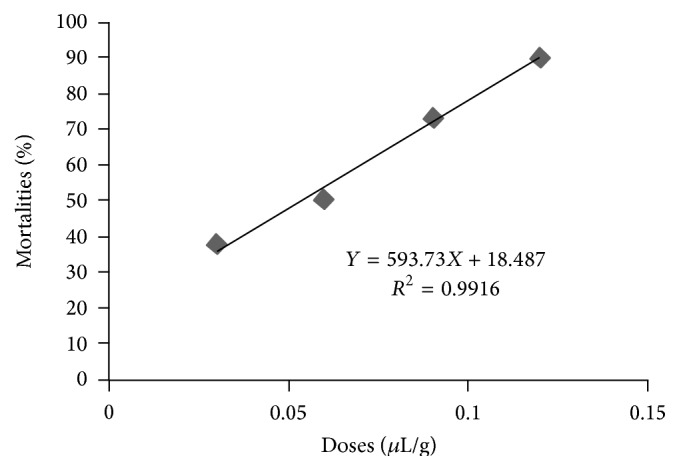
Evolution of mean cumulated percentages of* in vitro *mortality in terms of doses of the essential oils of* C. ambrosoides *contained in the soap.

The adjustment of cumulated mortality rates and corrected means [17] in terms of doses of the essential oil contained in the soap with time were used to draw the regression line (Figure 2):

(3) $$\begin{array}{c} Y=593.73X+18.487 \end{array}$$

(*R* ^2^ = 0.9916), where *Y* is the mortality rate and *X* is the doses of treatments. The high value of determination coefficient (0.99) showed that a large proportion (99.10%) of cumulated mortality rates could be attributed to the treatment.

The transformation of mortality rates into probits at the end of the second day in view of evaluating LC_50_ permitted us to obtain the probit regression line in terms of logarithm of doses of essential oil of* C. ambrosoides* in soap with the equation *Y* = 9.622*X* + 15.081 (*R* ^2^ = 0.9449), where *Y* is probit of mortality rate and *X* is the doses of treatments.

From this regression line, it is shown that, on the second day of exposure, LC_50_ is 0.037 *μ*L/g, thus confirming the high toxicity of essential oils of* C. ambrosoides *on* R. lunulatus*.

### 3.2. *In Vivo* Effect of the Soap Made from Essential Oils of* C. ambrosoides*

**Figure 3:**
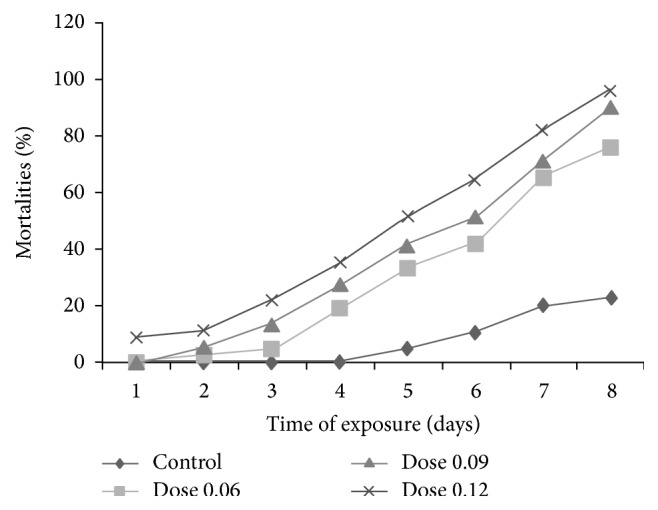
Variation of* in vivo* mortalities of* R. lunulatus* in terms of time and doses of the oil of* C. ambrosoides* leaves in soap.

The cumulated mortality rate increased with the dose of the oil and with time (Figure 3) to reach the highest level on day 8 (76.12, 90.27, and 96.29% for the doses 0.06, 0.09, and 0.12 *μ*L/g, resp.). Meanwhile, these mortalities were absent in the control for the first four days to reach the highest level of 22.69% on day 8.

The significant differences (*p* < 0.05) from day 2 observed among treated replicates with varied doses and between treated replicates and controls showed the increasing acaricidal effects of soap with time and depending on the doses of the essential oil of* C. ambrosoides* leaves in the soap.

The adjustment of the mean cumulated mortality rates [17]* in vivo* depending on doses of the essential oil with time gave a regression line with the equation *Y* = 269.67*X* + 13.847  (*R* ^2^ = 0.9963).

**Figure 4:**
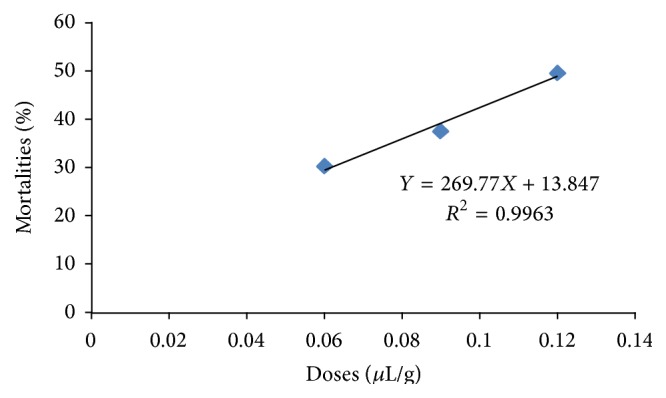
Evolution of mean cumulated percentages of* in vivo* mortalities in terms of doses of the essential oils of* C. ambrosoides* contained in the soap.

This regression line (Figure 4) indicated that a high proportion (99.63%) of the variation of cumulated mortality, corrected with time, is only due to the effects of different doses of essential oil contained in the soap. The transformation of mortality rates into probits at the end of the 2nd day in order to evaluate the LC_50_ helped us to obtain a regression line of probits of* in vivo* mortalities depending on the logarithm of doses of essential oil in the soap with the equation *Y* = 1.97*X* + 5.4448 (*R* ^2^ = 1). The LC_50_ derived from this equation was 0.059 *μ*L/g on the 2nd day of exposure, showing the* in vivo* toxicity of the soap made from essential oil of* C. ambrosoides* on* R. lunulatus.*

During this study, no undesirable effects were observed in the behavior of the animal.

Compared to the* in vitro* study, the mortality rates obtained* in vivo* were quite low. This difference may be due to fluctuations of experimental conditions. The undesirable effect of rain during treatments contributed to the washing off of chemicals applied on the animal. Other factors could be involved, as biotransformation of the active component by the animal's organs. Despite these remarks, it is obvious that soaps made from essential oil of* C. ambrosoides* leaves have toxic acaricidal effects on* R. lunulatus* in natural conditions.

## 4. Conclusion

In conclusion, it was observed that soaps from essential oil of* C. ambrosoides* leaves were toxic on* R. lunulatus* both* in vitro *and* in vivo*. Mortality rates of ticks increased gradually in terms of doses and time. This toxicity is not the same* in vitro* as* in vivo*. Low LC_50_ obtained (0.037 *μ*L/g* in vitro* and 0.059 *μ*L/g* in vivo*) showed the high toxicity of the applied treatment. The analysis of the chemical composition and the study of the acaricidal effects of the different fractions of the essential oil of this plant will permit us to improve on our knowledge of the toxic effects of our treatment. It will be equally important to determine with accuracy the active ingredient of the applied treatments and to study the safety of plant extract on the host animal.
